# Moderated Online Social Therapy: Viewpoint on the Ethics and Design Principles of a Web-Based Therapy System

**DOI:** 10.2196/14866

**Published:** 2019-12-04

**Authors:** Simon D'Alfonso, Jessica Phillips, Lee Valentine, John Gleeson, Mario Alvarez-Jimenez

**Affiliations:** 1 School of Computing and Information Systems University of Melbourne Parkville Australia; 2 Orygen Parkville Australia; 3 Centre for Youth Mental Health The University of Melbourne Parkville Australia; 4 School of Behavioural and Health Sciences Australian Catholic University Fitzroy Australia

**Keywords:** Web-based intervention, social network, well-being, eudaimonia, persuasive technology, ethical design

## Abstract

The modern omnipresence of social media and social networking sites (SNSs) brings with it a range of important research questions. One of these concerns the impact of SNS use on mental health and well-being, a question that has been pursued in depth by scholars in the psychological sciences and the field of human-computer interaction. Despite this attention, the design choices made in the development of SNSs and the notion of well-being employed to evaluate such systems require further scrutiny. In this viewpoint paper, we examine the strategic design choices made in our development of an enclosed SNS for young people experiencing mental ill-health in terms of ethical and persuasive design and in terms of how it fosters well-being. In doing so, we critique the understanding of well-being that is used in much of the existing literature to make claims about the impact of a given technology on well-being. We also demonstrate how the holistic concept of eudaimonic well-being and ethical design of SNSs can complement one another.

## Introduction

The modern omnipresence of social media and social networking sites (SNSs) brings with it a range of important research questions. One of these concerns the impact of SNS use on psychological well-being, a question that has been pursued in depth by scholars in the psychological sciences and the field of human-computer interaction (HCI) [1-5]. Despite growing multidisciplinary academic attention being devoted to questions concerning, for example, the “mental health outcomes associated with Facebook use” [6] over the course of the last decade, a related question concerning the design of social networking platforms remains underexamined, that is, *how do the design choices made by SNS programmers and related specialists contribute to the enhancement or the deterioration of a person’s psychological well-being?* It is the goal of this paper to discuss not only certain strategic design choices in our development of an enclosed social networking platform for young people experiencing mental ill-health but also to critique the understanding of well-being that is used in much of the existing literature to make claims about the impact of a given technology on well-being. We will argue that the conception of well-being used to evaluate the effects of a given technology is oftentimes limited and inconsistent and may impair rather than enhance our understanding of how SNSs impact individuals. We will demonstrate how the holistic concept of eudaimonic well-being and ethical design of SNSs can complement one another.

As researchers who study persuasive technologies and design SNSs for mental health, we recognize that we can contribute in playing “a watchdog role for the human computer interaction community in particular and the broader community” (p 230) [7] of consumers in general. This role includes the following 4 categories of action, as proposed by the persuasive technology pioneer Fogg [7]:

1. Identify artifacts and techniques: identify persuasive technologies and the persuasive techniques a researcher uses.
2. Examine effectiveness and effects: assess the effectiveness (intended impact) and the effects (unintended side effects) of persuasive technologies or strategies.
3. Disclose findings: those who study persuasive technologies have an obligation to disclose their findings.
4. If necessary, take or advocate social action: if a computer or artifact is deemed harmful or ethically questionable, a researcher should either take social action or advocate that others do so.

We will begin this paper by defining the term *social network* before proceeding with an overview of persuasive technology design and some associated ethical issues. Next, we will discuss the tendency in the existing literature to evaluate the effects of a given technology in relation to a limited concept of well-being that aligns with what researchers term *subjective well-being* (SWB) or *evaluation well-being* [8]. We will argue that the concept of SWB tells us very little about “what constitutes a well-lived life” [9] and, in turn, provides us with a narrow understanding of how SNSs impact individuals. Huang emphasized these difficulties in a 2010 meta-analysis, observing across 40 studies that well-being was inconsistently represented “by numerous psychological constructs, such as loneliness, depression, self-esteem, and life satisfaction,” making it very difficult to draw any conclusions across the studies and contributing to mixed findings regarding the topic [10]. In this section, we will contrast *SWB* with what Ryff terms “a eudaimonic approach to psychological wellbeing” [9] and argue that the depth and breadth of this concept make it a valuable framework to evaluate the effects of an SNS platform and to guide the design and development of SNSs in youth mental health in particular. From here, we will provide an overview of the Moderated Online Social Therapy (MOST) platform we have developed and analyze it in terms of ethical persuasive design and eudaimonic well-being before outlining some new developments and discussing them in terms of the themes of this paper. Later topics covered include how MOST promotes a balance in online/offline activity and the incorporation of gamification.

The aims of this paper are in line with Fogg’s categories of action as outlined above, focusing primarily on the first 2, that is, identifying artifacts and techniques that may render our technology persuasive and examining their effects and effectiveness, which involves assessing both the intended and unintended effects of our strategies. Disclosing our findings and advocating social action are, incidentally, actions performed in the writing and dissemination of this paper.

## What Is a Social Network Site?

Scholars Boyd and Ellison differentiate between a social network site and a SNS. They define a social network site as [11]:

A web based service that allows people to 1) construct a public or semi-public profile within a bounded system; 2) articulate a list of others users with whom they share a connection; 3) view and traverse their list of connections and those made by others within the bounded system.

They prefer the term social *network* site because these Web-based platforms are primarily designed to “enable users to articulate and make visible their [existing] social networks” (p 211) [11]. Put differently, they are not about making new friends or networks but about tending to their existing ones.

Networking, on the contrary, “emphasizes relationship initiation, often between strangers” (p 211) [11]. It is in this sense, however, that MOST is an SNS. Thus, adapting from Boyd and Ellison’s definition, MOST is an SNS best conceived as a Web-based service that allows people to construct a public or semipublic profile within a bounded system and to initiate relationships generally between strangers. The vast majority of users who join the MOST SNS platforms for mental health do not know each other before use. Therefore, one of the primary goals of the interventions is for users to connect with others who share a similar lived experience but do not necessarily share a similar or the same offline social networks.

## Persuasive Technology Design

The study of computers as persuasive technologies has roots in Fogg’s seminal work and his coinage of the term *captology* from the acronym Computers as Persuasive Technologies [7,12]. According to Fogg, “a persuasive computing technology is a computing system, device, or application intentionally designed to change a person’s attitudes or behavior in a predetermined way” [13]. Work in the field of persuasive technology design is particularly useful as a foundation for the development of digital technologies for behavior change and psychological well-being (PWB). Informed by Fogg’s conceptualization of persuasive technology, Oinas-Kukkonen and Harjumaa [14] have developed a more concrete framework that transforms persuasive design principles into software requirements and system features. According to their Persuasive Systems Design model, there are 4 categories for persuasive system principles:

1. Primary task: The design principles in this category support the carrying out of the user’s primary task and consist of reduction, tunneling, tailoring, personalization, self-monitoring, simulation, and rehearsal.
2. Dialog: The design principles in this category are about the feedback an interactive system provides to its users to help them move toward their goal or a target behavior. This category consists of praise, rewards, reminders, suggestion, similarity, liking, and social role.
3. System credibility: The design principles in this category describe how to design a system so that it is more credible and thus more persuasive. The category consists of trustworthiness, expertise, surface credibility, real-world feel, authority, third-party endorsements, and verifiability.
4. Social support: The design principles in this category describe how to design the system so that it motivates users by leveraging social influence. The category consists of social facilitation, social comparison, normative influence, social learning, cooperation, competition, and recognition.

In his earliest expositions of captology, Fogg brought attention to the fact that “adopting an ethical perspective in this domain is vital because the topic of computers and the topic of persuasion both raise important issues about ethics and values” [7]. In the environment of the World Wide Web, social media, and ubiquitous personal devices such as smartphones, this topical theme takes on pressing significance.

Motivated primarily by commercial and advertising interests, many websites and apps incorporate features that are intentionally designed to hook users in, maximizing their attention and usage time without due regard for the quality or benefits to well-being of this usage. A movement has recently emerged to counter this phenomenon, colloquially termed *brain hacking*, by promoting the development of technology that is ethical and humane rather than addictive or of little genuine benefit. Founded by Tristan Harris, a former student of Fogg, the Center for Humane Technology (CHT) [15] is raising awareness of the issues associated with certain SNSs and smartphone technologies. The CHT writes that [16]:

Facebook, Twitter, Instagram, and Google have produced amazing products that have benefited the world enormously. But these companies are also caught in a zero-sum race for our finite attention, which they need to make money. Constantly forced to outperform their competitors, they must use increasingly persuasive techniques to keep us glued. They point AI-driven news feeds, content, and notifications at our minds, continually learning how to hook us more deeply—from our own behavior.

The CHT is “creating humane design standards, policy, and business models that more deeply align with our humanity and how we want to live” [16]. The establishment of app design guidelines offers a simple and effective way to influence app architects by positively shaping the design and development of their systems. For example, the following is a selection of pertinent principles taken from a list published by the CHT that have been applied to the MOST platform [17]:

1. Does your product honor both on- and off-screen possibilities?
2. Does your product enhance relationships or keep people isolated?
3. Does your product land specific, net positive benefits in people’s lives?
4. Does your product eliminate detours and distractions?

In our subsequent discussion of the MOST system, we will examine how it adheres to or embodies such persuasive design principles. Let us now discuss how well-being is traditionally conceptualized in the existing literature and provide the rationale for why we have chosen to evaluate the MOST system in terms of a eudaimonic conception of well-being.

## What Are We Talking About When We Talk About Well-Being?

In a systematic review on the “Impact of the Use of Social Network Sites on Users’ Psychological Wellbeing,” Erafni and Abedin [8] argue that research on *psychological well-being* has historically been aligned with 3 perspectives: the hedonic view, the eudaimonic view, and life satisfaction (also known as the evaluation well-being view). The hedonic and evaluation well-being views are frequently referred to as PWB in the literature and are rarely differentiated as different *kinds* of well-being. However, these distinctions, which we will discuss in a moment, are nontrivial, for when several measures of well-being are grouped beneath the broad umbrella term PWB, it becomes difficult to understand precisely *what* is impacted by a given technology.

SWB [18] concerns “moods and emotions [...] together labelled *affect* [and] represents people’s on-line evaluations of the events that occur in their lives” (p 277) [19]. SWB consists of 3 components: “life satisfaction, the presence of positive mood, and the absence of negative mood, together often summarized as happiness” (p 144) [20]. Ryan and Deci argue that aligning well-being with happiness has a long history, dating back to the Greek philosopher Aristippus in the fourth century Before Christ. Thomas Hobbes, DeSade, and Jeremy Bentham followed in Aristippus’s thinking and later enlarged this early philosophical hedonism [20]. The prevailing view present among hedonic psychologists today, Ryan and Deci argue, is the idea that well-being “consists of subjective happiness […] the experience of pleasure versus displeasure broadly construed [and] all judgements about the good/bad elements of life” (p 144) [20]. To illustrate how prevalent the concept of SWB is in discussions of SNSs, Erafni’s and Abedin’s analysis indicates that of 22 studies that met their criteria for inclusion, 15 used the measure of life satisfaction, 3 used the measure of affect or happiness, and 1 used the components we normally associate with eudaimonic well-being: autonomy, personal mastery, personal growth, positive relations, purpose in life, and self-acceptance [8]. However, it has been debated whether the SWB construct offers an adequate evaluation of a person’s psychological wellness [21,22].

Carol Ryff has argued that prevailing concepts of SWB must be challenged as the construct’s narrow focus on “assessments of feeling good, contentment and life-satisfaction” (p 13) [21] neglects “aspects of positive functioning such as purposeful engagement in life, realization of personal talents and capacities, and enlightened self-knowledge” [22]. Self-Determination Theory (SDT) pioneers Ryan and Deci have also challenged the SWB construct of well-being through their adoption of the concept of eudaimonia in their broader consideration of well-being and “what it means to actualize the self and how that can be accomplished” (p 146) [20]. SDT recognizes that 3 fundamental psychological needs are essential for the fulfillment of psychological growth. These include the need for autonomy, competence, and relatedness. For SDT, the fulfillment of these 3 basic needs is both the “natural aim of human life” and “typically fosters SWB as well as eudaimonic wellbeing” (p 147) [20]. In fact, a recent study on designing digital systems for motivation, engagement, and well-being shows how satisfying the 3 basic needs of SDT can increase these desired outcomes of user experience [23].

Ryff argues that SWB surfaced because of a mistranslation of the Aristotelian concept of *eudaimonia.* She writes that *eudaimonia*, when translated by Bradburn and other utilitarian philosophers of the 19th century, was taken to mean happiness [21]. Ryff argues that the trouble with this assumption is that it equates hedonia with eudaimonia, “something that was deeply contrary to Aristotle’s distinction between the satisfaction of right and wrong desires” [21]. Furthermore, this conflation leaves out the essence of Aristotle’s eudaimonia: “the striving towards excellence based on one’s unique potential” [21]. Ryan and Deci too argue that Aristotle thought hedonic happiness and the pursuit thereof to be a *vulgar idea*. In their view, Aristotle believed that *true happiness* was to be found in “the expression of virtue” or “in doing what is worth doing” [20]. Therefore, Ryff’s project has been to articulate a conception of PWB that is informed by and aligned with what she perceives to be the original essence of Aristotle’s *eudaimonia.* Ryff’s concept of PWB, the construct on which we will base our assessment of the MOST digital intervention for mental health, is concerned with 6 core components: self-acceptance, autonomy, personal growth, positive relationships, environmental mastery, and purpose in life [21].

It is evident then that SWB and PWB, although both aimed at understanding the greater question of what makes a good life, are underpinned by radically different value judgments about what that is. From the hedonic viewpoint of SWB, well-being is “equated with happiness and is formally defined as more positive affect, less negative affect and greater life satisfaction” [20]. PWB or eudaimonic well-being, as characterized by Ryff’s construct, in contrast, conceives of well-being as a broad and complex concept consisting of 6 intersecting variables (as above) and has little to do with the pursuit of pleasure and a diminishment of negative affect. PWB recognizes that the pursuit of well-being is frequently characterized by periods of negative affect, especially when one pursues a meaningful and difficult goal.

What this discussion of SWB and PWB reveals is that the construct of well-being is *controversial and unresolved* [20]. For that reason, it is less than straightforward to interpret claims made about how technology impacts individual well-being. Furthermore, the overreliance of SWB measures to assess the impact of technology suggests that we are ascertaining a very narrow understanding of technology’s impact. An additional point worth emphasizing is that psychology as a discipline has for the last century been concerned with “the amelioration of psychopathology” and not with the enhancement of well-being and individual growth [20]. Therefore, PWB is central in our discussion of the nexus between mental health and SNSs because the focus for psychology as a discipline has shifted, and we are now witnessing an increased focus on recovery in the fuller sense, including social and economic participation. We want to now bring these focal changes to bear on SNS design in particular. In the section to follow, we turn to a discussion of the MOST system in terms of ethical persuasive design and well-being considerations, showing how MOST fosters this broader sense of PWB or eudaimonic well-being.

## Moderated Online Social Therapy

The MOST project, based at eOrygen (the digital mental health division of Australia's Orygen youth mental health centre), has been researching and developing online social therapy systems for mental health since 2010. Consisting of a multidisciplinary team of clinical psychologists, computing and information systems researchers, software developers, creative writers, illustrators, and peer workers, MOST has been primarily concerned with evaluating the efficacy of online therapy and developing engaging digital technologies for young people experiencing mental ill-health. Trials have also been adapted to nonyouth cohorts, specifically the families/carers of such young people.

The result of this work has been an online social therapy framework powered by the MOST Web platform (or MOSTware), which integrates Facebook-style social networking, specialized therapy units, and a forum-like feature where users can pose and cooperatively crowdsource solutions to common problems [24-26]. All of this occurs within a clinical and peer-moderated environment.

As will be detailed later on, the MOST framework has been inspired by the field of positive psychology. Thus, on this basis, it technologically embodies an approach that balances a traditional focus on psychopathology with an emphasis on positive human development and flourishing [27,28]. Together with a participatory design approach involving the users of our systems (young people and their carers) [29], “we became aware of evidence highlighting that a focus on deficits in online systems can lead to demoralization and disengagement and so we began to specifically draw upon positive psychology frameworks” [30]. More generally, MOST, as a technology that supports well-being and flourishing, aligns with the field of positive computing, which is itself informed by positive psychology and provides a foundational framework for the “design and development of technology to support psychological wellbeing and human potential” [31].

By tailoring therapy content to target the treatment of specific conditions and adding any required code customizations, the flexible MOST platform enables the setting up of individual sites for a variety of mental health cohorts. To date, MOSTware has powered several successful studies, including the following:

- Rebound, a pilot trial for relapse prevention of major depressive disorder in young people [32].
- Meridian, a pilot trial for carers of young people diagnosed with depression and anxiety [30].
- Momentum, a pilot trial for young people at ultrahigh risk for psychosis [33].
- Altitudes, a 2-year randomized controlled trial (RCT) of MOST for carers of young people with psychosis [34].
- Horyzons, a 5-year RCT of MOST for relapse prevention following a first episode of psychosis [35]. This trial will be used throughout this paper for illustration of the MOST platform.
- Generation, a public trial for general help-seeking youth, in collaboration with the eheadspace [36] electronic mental health service [37].

System credibility (as described in the section Persuasive Technology Design) within these interventions is ensured by several factors and measures taken:

1. Therapy content is created by expert psychologists, young people with a lived experience of mental ill-health, writers, and illustrators.
2. System moderation is conducted by qualified clinical psychologists and youth workers.
3. Peer moderation is conducted by young people with a lived experience of mental ill-health who model system usage and interact with clients.
4. Clinical trials are conducted with established protocols and within a framework of formal ethics approval.

### Social Network

**Figure 1:**
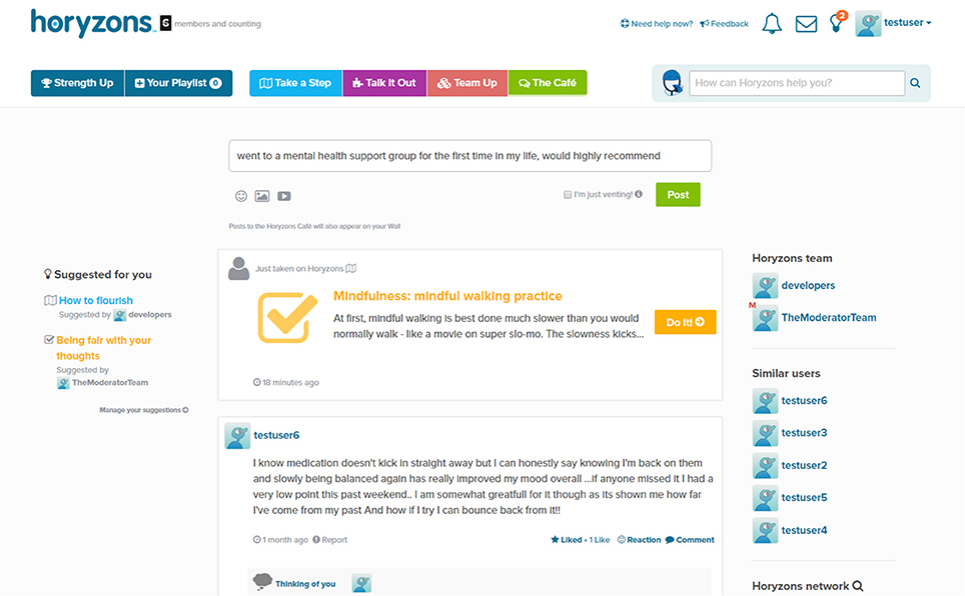
The Moderated Online Social Therapy news feed.

In the social network newsfeed section of the system otherwise known as The Café (Figure 1), users can contribute posts and comments, share experiences and interests, give and obtain support, gain new perspectives, and search for job opportunities and information in the Job Zone. The newsfeed feature is such that all users see the same newsfeed (ie, same ordered list of posts), though new developments will see the introduction of an algorithmically driven newsfeed tailored to each individual user. In Team Up, a relatively new feature to be discussed in more depth later, users can set a challenge for themselves that others can also participate in or follow as members of a *cheer squad*. MOST also contains an instant messenger service for communication between users, though this is only activated in the social network section of the site so as not to distract users with message popups while they are engaging with other parts of the site, particularly therapy content that requires deep attention. This design choice aligns with the CHT principle concerning the elimination of product detours and distractions.

### Steps and Actions

**Figure 2:**
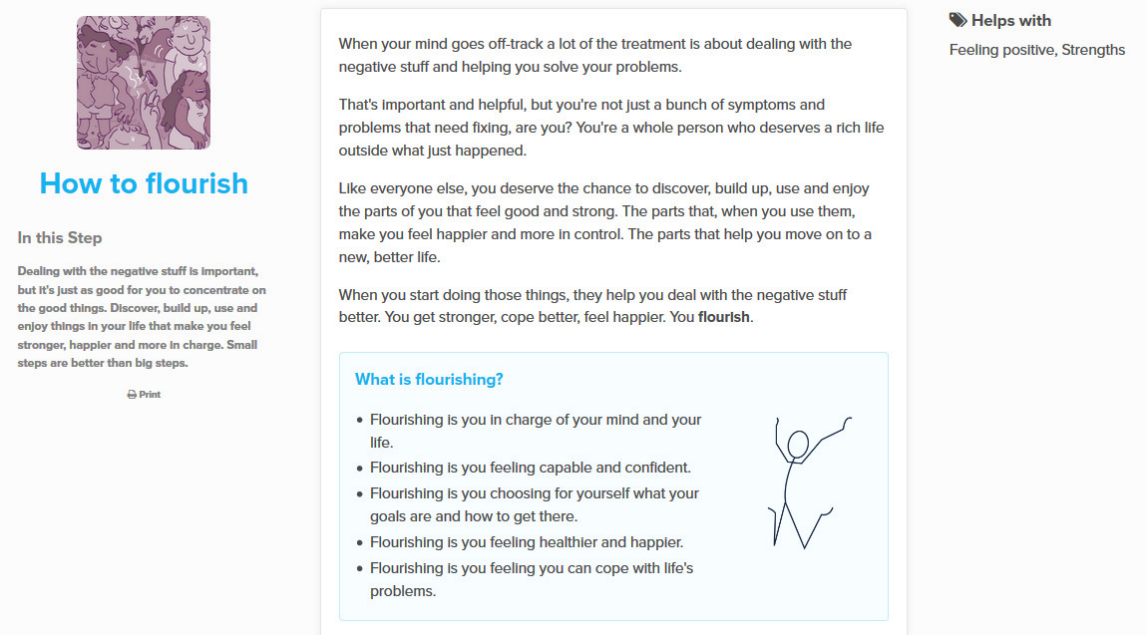
The Step How to flourish.

The 2 types of therapy units offered in the MOST system are *Steps* and *Actions*. Steps are psychoeducational therapy modules that assist people with developing psychological skills, such as self-compassion and mindfulness, and core components of eudaimonic well-being or PWB, such as purpose in life, autonomy, personal growth, environmental mastery, positive relationships, and self-acceptance. Although there is still a case to be made for helping users to regulate unnecessary and pointless suffering by reducing or moderating unhelpful and oftentimes distressing emotional states, the ultimate aim of Steps and Actions is to foster PWB, particularly in relation to autonomy and self-acceptance. These standalone therapy units can also be found and sequentially completed within themed Step collections called *Pathways*. Steps are delivered as engaging content, including low-literacy comics, and have been developed collaboratively by clinical psychologists, creative writers, young people, and graphic designers. In terms of dialog support [14], as introduced in the section Persuasive Technology Design, therapy content has been developed to imitate its users with relatable material and suitable language for the target cohort (similarity) with an appropriate look and feel (liking). Figure 2 illustrates an example Step, *How to flourish*, which is primarily concerned with supporting young people to locate purposeful activities and meaning in their lives.

**Figure 3:**
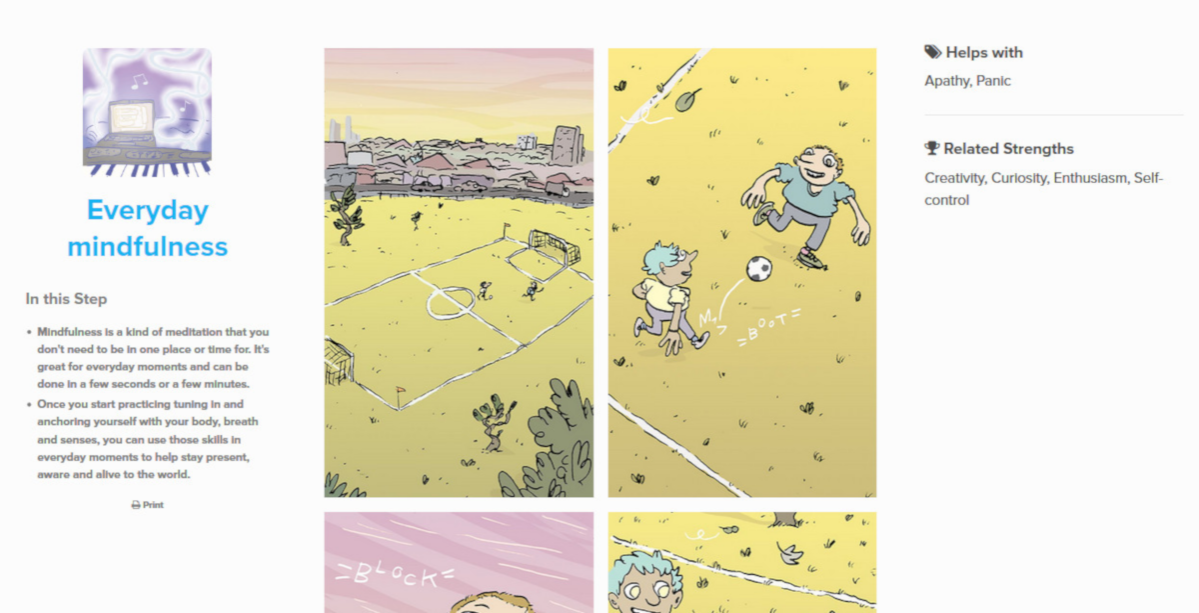
The Step Everyday Mindfulness.

Figure 3 illustrates part of another example Step, *Everyday mindfulness*, in the newer comic format.

**Figure 4:**
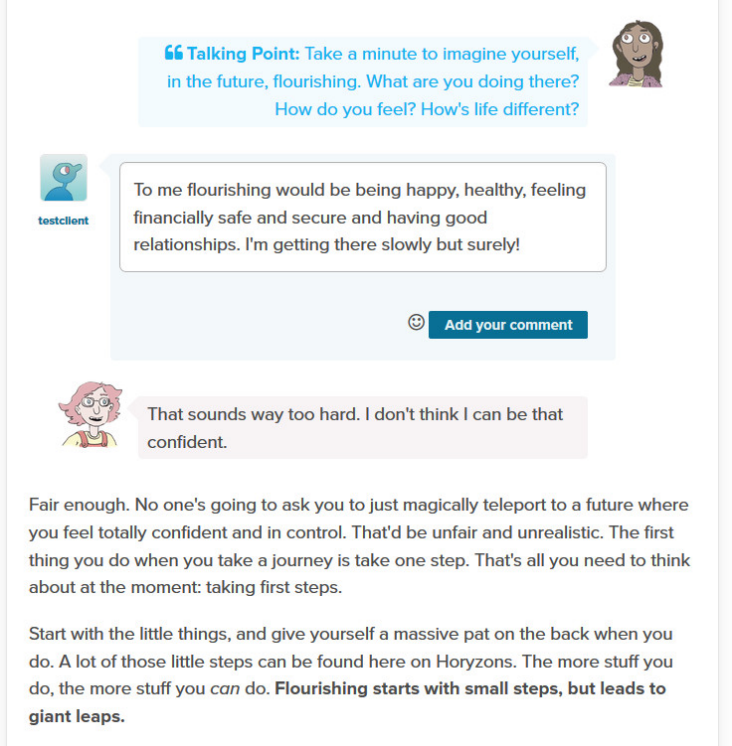
A Talking Point for the Step How to flourish.

Social interaction is embedded within Steps through Talking Points, which are focused questions that invite young people to discuss and share their own experiences. Figure 4 shows a Talking Point within the Step *How to flourish*.

**Figure 5:**
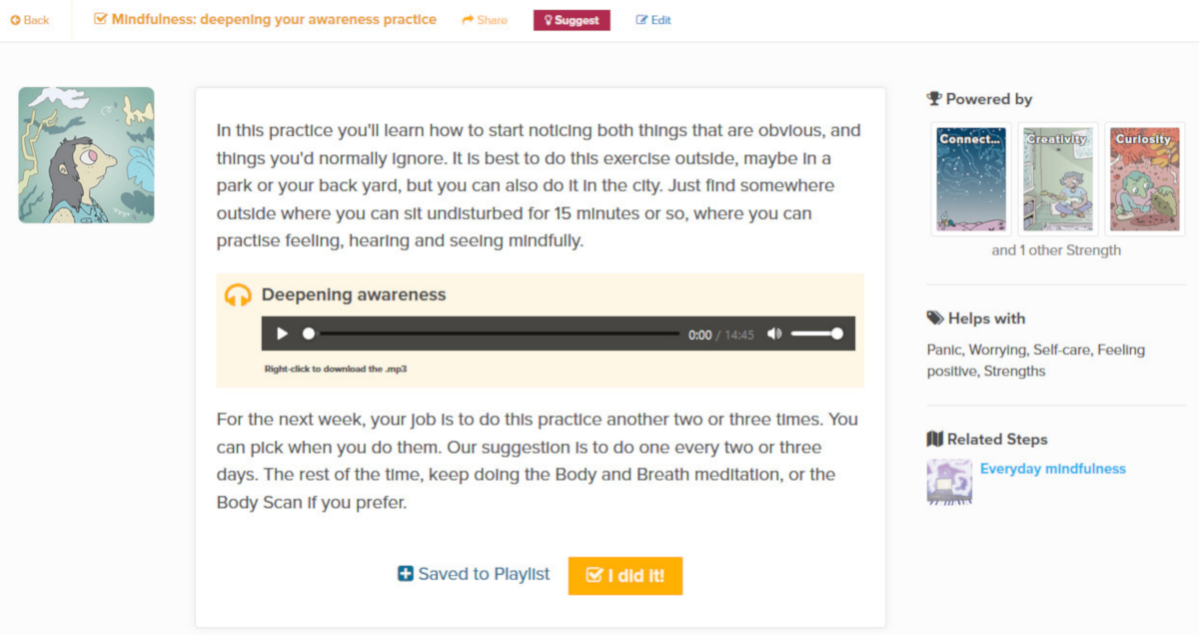
The Action Mindfulness: deepening your awareness practice.

Actions are bite-sized behavioral activities or tasks that users can do to apply mindfulness, self-compassion, and personal strengths to relevant real-world situations. The use of context-specific, action-based suggestions through online interventions has been recommended to change behavior, develop skills, and increase practice and generalization of these skills to real-life situations [38]. Users are responsible for completing the action in their own time and are encouraged to report back to others with their progress. Through choosing relevant and personally meaningful actions, the intention is that users come to recognize that they have the capacity to shift things in their lives for the better. Put differently, actions aim to foster a sense of environmental mastery through autonomy by urging users to recognize and indeed experience the degree of control they have over many of the circumstances of their lives. Certain Actions are embedded within the Steps to which they are relevant, so that once a Step is completed, users can do an Action to reinforce what they have learned (Figure 5 shows an Action that is embedded in the Step *Everyday mindfulness*). Users can also engage with Actions through the Strengths to which they are connected; if an Action is connected to a Strength, then doing that Action will help to promote that Strength.

### Strengths

**Figure 6:**
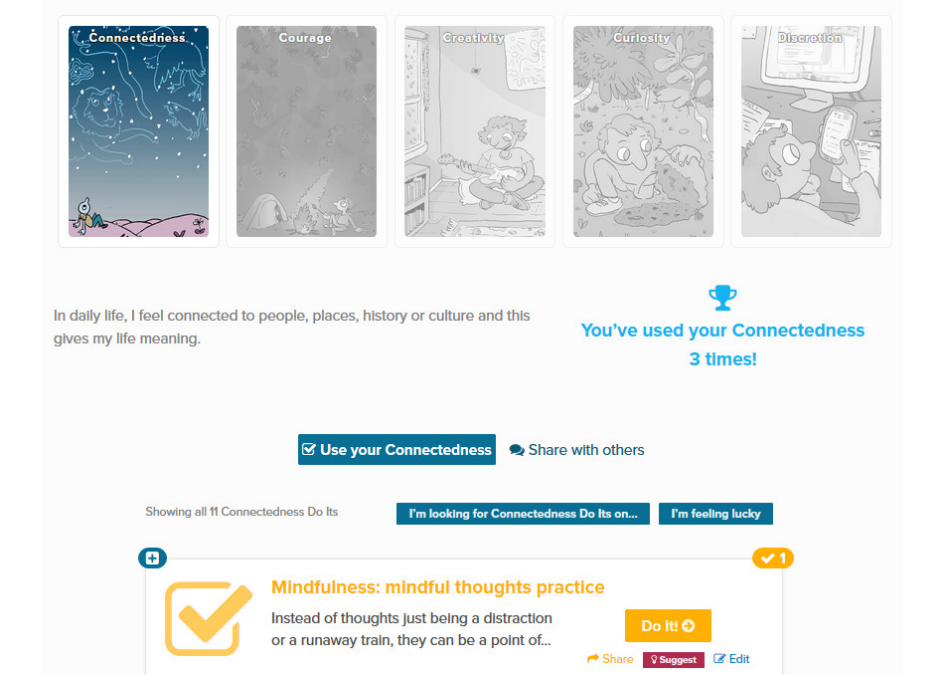
The Strengths page.

Upon joining the system, users have the option to complete an initial exercise where they choose 5, out of 24, character strengths that they believe best apply to them. Although they are free to partake in activities associated with any of the Strengths, content associated with the Strengths a user selects will be promoted by the MOST system and used to guide the user through the experience over time. An illustration of the Strengths page after completion of this initial exercise is given in Figure 6.

The set of Strengths available in the MOST platform is not fixed and can be customized according to the purpose of a particular intervention. The most representative set of Strengths, found in interventions such as Horyzons, consists of 24 strengths drawn from the field of positive psychology [39]. In addition to those illustrated in Figure 6, others include fairness, forgiveness, gratitude, and honesty.

Connected with the ideas and frameworks discussed in the earlier section on well-being, the positive psychology movement is defined as “the scientific study of positive human functioning and flourishing on multiple levels that include the biological, personal, relational, institutional, cultural, and global dimensions of life” [28]. A complement rather than replacement for traditional areas of psychology, positive psychology is concerned with human flourishing, a doctrine where the concept of eudaimonia, that is, “the striving towards excellence based on one’s unique potential” [21], is deemed central to a life lived well. Positive psychology understands the good life as that in which one uses their “signature strengths every day to produce authentic happiness and abundant gratification” [40]. The 24 Strengths drawn from positive psychology and embedded in the MOST system correspond neatly with the building blocks of eudaimonic well-being. By encouraging users to identify strengths they currently possess and those they wish to build, the system strives to promote self-acceptance, self-competence, and personal growth. For instance, users come to recognize and identify their current positive attributes (self-acceptance). An invitation that may be infrequently offered in the offline world, users also come to value personal growth through the recognition that their personal circumstances are not fixed; rather they can indeed *grow* or develop strengths that they do not currently believe they possess.

Thus, the positive psychology–inspired Strengths and therapy features of MOST naturally align with eudaimonia and Ryff’s concept of PWB. Although MOST interventions strive to maintain or improve levels of SWB, they also ultimately aim to promote eudaimonic or PWB.

### Talk It Out

The Talk It Out (TIO) feature is a space where users can nominate problems or difficulties that they are seeking to discuss with other users. TIO is based on an evidence-based cognitive behavioral therapy social problem-solving framework developed by Nezu et al [41]. Once a user has nominated a problem and framed it together with a peer worker, they brainstorm solutions together with others and discuss the pros and cons of each suggestion. Those moderating the process then synthesize the content and *wrap it up*. Apart from the role each of these TIO discussions play at the time in providing an active forum to help a person navigate their way through a problem, the overall result is an invaluable user-generated *knowledge base* repository that can be searched and referred back to by users at any time.

Environmental mastery is perhaps the most pertinent aspect of eudaimonic well-being fostered by TIO. Through the various phases, participants learn how to approach problem solving in a *systematic and thoughtful* way [41]. They learn how to clearly and unemotionally define the problem they are facing, and in doing so, they are better positioned to ask for help and others are more likely to provide help that is relevant to their particular problem. The second phase of the TIO, brainstorming, further contributes to a sense of environmental mastery as it helps participants to recognize that when they perceive that there are multiple ways to solve a well-defined problem, they become less stressed and feel more control over their lives, safer, and more hopeful about locating a solution [41]. Finally, brainstorming contributes to a sense of environmental mastery by inviting people to externalize their problem [41]. Thus, rather than ruminating on their own thoughts, brainstorming removes the isolation that may accompany an internalized problem and supports participants to recognize that by externalizing a problem to others (or a network of other people like them) they can feel more in control of locating a solution.

In line with the CHT guidelines listed in section Persuasive Technology Design, TIO has been developed to foster connectivity and to help counter isolation while also promoting a sense of autonomy. The TIO feature is nonhierarchical—there is no patient and there is no expert. Rather, all participants are invited to be the experts on their own lives. This setup aims to foster a sense of autonomy as it removes the need to defer to a clinician when seeking a solution to an everyday problem. Through the TIO, participants can come to recognize that their lived experience is valuable and can be used, whether they are in a formal role as a peer worker or not, to support others. Therefore, TIO should bring people together and empower them to both contribute to and seek social support.

Unlike other social media and networking sites, the MOST platform has been specifically developed for the online treatment of mental ill-health. Naturally, therefore, a paramount consideration is the clinical efficacy and HCI of its specialized system components, such as the TIO forums, Steps and Actions therapy units, and moderator involvement. However, beyond these technical and clinical considerations and in concert with them, we must be mindful of the following:

1. Ensure that the system in general is not detrimental to well-being and that features and components of little value or those that have a negative impact on psychological health are avoided.
2. Develop content and features that promote mental health and well-being and that users are motivated to use for good reasons.

In describing the MOST system throughout this section, we have covered how some of these considerations have been incorporated into its design. We now turn to discuss some new MOST developments as they relate to the themes of this paper.

## Balance Between Online and Offline Activities

Commercial SNSs often aim to maximize their sphere, not only in terms of user attention and usage time but also in terms of bringing and containing as much activity as possible within the site. Contrary to such monopolization, we maintain the following:

1. A website should encourage and facilitate offline activity outside of the site.
2. Online activity should be promoted within the site insofar as it is something that is beneficial and inherent to the site or can genuinely only be mediated/facilitated by the site.

This approach ties in with the CHT design principle “does your product honor both on and off-screen possibilities” and helps to deter social media and networking addiction [42,43]. We posit that it also accords with the eudaimonic conceptualization of well-being in the sense that the good life cannot be one confined to the digital realm. The resulting design philosophy is embodied in several existing and upcoming features and functionalities associated with the MOST system.

### Team Up

**Figure 7:**
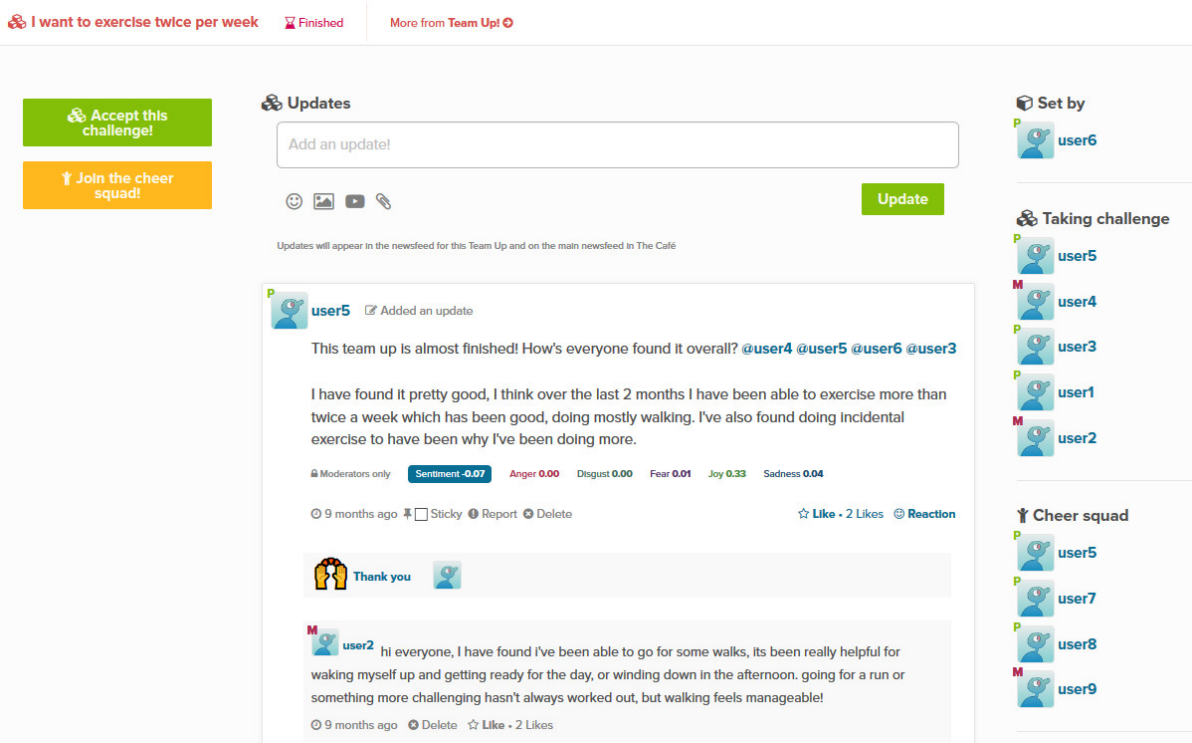
An example Team Up challenge.

The *Team Up* feature is a relatively recent addition to the MOST platform, and a minimum viable implementation has been tried out on a select few trials. Team Up begins with a user nominating a challenge or goal that they want to achieve by a certain date. Other users can join the challenge either as a fellow participant or a supportive follower. An example Team Up challenge, “I want to exercise twice per week,” is captured in Figure 7. As can be seen, Team Up embodies several social support persuasive system principles [14]:

1. Social learning: A person will be more motivated to perform a target behavior if (s)he can use a system to observe others performing the behavior.
2. Social facilitation: System users are more likely to perform target behavior if they discern via the system that others are performing the behavior along with them.
3. Recognition: By offering public recognition for an individual or group, a system can increase the likelihood that a person/group will adopt a target behavior.

However, other social support design principles such as competition and normative influence or peer pressure seem inappropriate given the type of supportive atmosphere being fostered in MOST.

Team Up demonstrates a commitment to ensuring that the systems strike a balance between online and offline activity. This simple feature drives an activity loop whereby users initiate their intention within the MOST system, and they then proceed to carry out activity in the offline world. They then return online to the MOST system to report their progress, and this cycle can continue. Crucially, without some activity in the offline world, there is no point in using the online feature, and thus, it encourages healthy activity offline, promotes social support and interaction within the online community, and uses the site only to facilitate these 2 things. Support and validation are offered to the Team Up challenger by other challengers and supporters joining, and this in turn promotes and encourages the completion of a beneficial goal. Interestingly, in a review of persuasive design in internet-based psychological therapy systems for adolescent depression [44], it is claimed that “persuasive design features that leverage social support to motivate users were rarely, if ever, reported features.” Such opportunities should not be missed, and the Team Up feature is one such example.

### Online and Offline Therapy

The complete therapy experience in MOST consists of both online and offline units. Steps, which can be considered the digital counterpart to traditional face-to-face therapy, are engaged with and contained within the online MOST platform; users read the content and rate, share, and comment on screen.

However, Actions, which are often embedded within the Steps they are related to, generally involve doing an offline exercise to reinforce skills, especially those covered in the Step. Once a Step is completed by the user online, relevant Action suggestions populate the bottom of the Step screen, thus offering *choices that send the user off the screen*, as per the guidelines offered by the CHT.

The fact that Actions are embedded and confined within the MOST platform does, however, present a certain limitation to their utilization. This is because although access to the library of Actions is presently confined to the MOST Web app via standard access points such as lists and a search bar, they generally involve activity that is done offline and outside of the platform in relevant real-world scenarios. To remove these online to offline barriers and facilitate Action engagement, a new Action delivery system in the spirit of just-in-time adaptive interventions is in development [45]. The idea is to respectfully use smartphone sensors to determine information about a user’s present psychological states and/or their current situation or location and then use this information to deliver to them, using push notifications contextually relevant and helpful Actions they can do in situ, in real time [46].

For example, the Action “Mindfulness: deepening your awareness practice” (Figure 5) contains an audio guide on mindfulness with the suggestion that it be done outside in a park. Mobile phone geolocation data could be used to determine if a user is spending time in a park and deliver such an Action suggestion. As another simple example, the detection of a bout of nocturnal phone activity that deviates from one’s standard usage times or is at odds with their indicated chronotype could lead to the suggestion of an exercise for insomnia.

Given such a system, a user could gain therapy benefits without having to log into the MOST platform. The aim is for such a system to reduce entry barriers into therapy completion and increase motivation and the number of Actions a user fulfills. Thus, beyond simply offering and expecting users to visit a website for help, the system can meet users in real-life situations and dynamically deliver accessible and effective personalized help directly to their pockets. In terms of the persuasive system principles by Oinas-Kukkonen and Harjumaa [14], this mobile therapy delivery system embodies the primary task support principles of tailoring (information provided by the system will be more persuasive if it is tailored to the potential needs, interests, personality, usage context, or other factors relevant to a user group) and personalization (a system that offers personalized content or services has a greater capability for persuasion).

Such a detect-and-deliver system may also inhibit detrimental smartphone usage. In their article on 10 lessons learned about SNSs and addictions, Kuss and Griffiths (p 8) [43] list “Smartphone Addiction May Be Part of SNS Addiction” and write the following in their discussion of this item:

According to the pathway model, an addictive pattern of mobile phone use is characterized by the use of specific applications, including calls, instant messaging, and the use of social networks. This suggests that rather than being an addictive medium per se, mobile technologies including smartphones and tablets are media that enable the engagement in potentially addictive activities, including SNS use.

In terms of this issue, the benefit of such a detect-and-deliver system is that the smartphone becomes a tool to deliver contextually relevant Actions suggestions in offline situations rather than a necessary portal to an online app.

## Gamification

Gamification, as standardly defined, is the use of “video game elements in nongaming systems to improve user experience (UX) and user engagement” [47]. Not surprisingly, there is an overlap between gamification and persuasive technology [48]. Gamification ties in with the Dialog Support Rewards principle (systems that reward target behaviors may have great persuasive powers) [14], and “some persuasion mechanisms can be regarded as similar to those applied in gamification, such as feedback and rewards” [49]. There are also certain ethical dimensions of gamification to consider, including the argument that gamification is at odds with human flourishing and that it could be “morally corrosive by adversely impacting character” [50]. There is currently work being done on adopting gamification to promote (mental) health and well-being [51-54]. We see the potential to positively harness gamification to enhance the adoption and sustained use of persuasive technologies that promote positive behavior change for people, particularly youth, in the mental health sphere.

Although not a formative consideration during the initial design and development of the MOST system, developments have since started to incorporate some gamification to encourage social engagement and therapy participation. This is in part because of feedback from users who have expressed a desire for the incorporation of gamification, particularly as ascertained from recent posttrial interviews for a qualitative study in development. In designing gamified components, we recognize that it is important to ensure that gamification does not negatively impact users and is not exploited by other parties. When done appropriately (see the studies by Kim and Werbach [55] and Llagostera [48] for an overt example of inappropriate and pernicious gamification), competitiveness may be a suitable and effective ingredient in a gamified system. However, given that MOST is designed for young people experiencing a variety of mental health conditions, they may be particularly vulnerable or sensitive to potential negative effects of inciting competition and social comparison. Thus, the focus is on personal gamification and opportunities to make gamification a social, but not necessarily competitive, experience. In game theoretical parlance, we are dealing with non-zero-sum, possibly cooperative, games.

As introduced in the section Moderated Online Social Therapy upon joining the system, users can complete an initial exercise where they choose 5 out of 24 Strengths that they believe best apply to them. Changes to the Strengths system, at this stage in prototype form, involve adding a gamification component. Rather than a user selecting a fixed group of their top-5 Strengths when they begin using the site, with this new system, each Strength starts out as being more or less equal and various bits of user activity in the site over time add to the points score that they achieve for each strength. For example, contributing a newsfeed post could add 1 point to a user’s Social Connectedness strength score. As another example, users could gain Strength points in their Supportiveness strength by contributing a response to another user’s TIO problem.

The TIO feature is particularly interesting in terms of gamification considerations. As described earlier, the basic flow of a TIO discussion starts with the suggestion of a problem for communal discussion. Once this problem has been shaped, brainstormed solutions are offered by participants. A list of established solutions is then discussed by the group, where for each solution offered, users can respond to the solution with commentary responses in the form of *pros* and *cons*. Unlike the MOST news feed, in which posts and comments can be responded to via likes and reactions, no such response options to user posts have been implemented in TIO. The forum-like, crowdsourced question-and-answer structure of TIO makes it amenable to a style of reputation system gamification, in a manner similar to that of highly popular crowdsourcing forums such as Stack Overflow, where users are incentivized to contribute responses to questions by a points-and-recognition system. Users earn reputation points for offering answers, having their answers upvoted or downvoted by peers, and can receive badges for their valued knowledge contributions [56-58].

Despite the suitability and tremendous success of Stack Overflow’s gamified reputation system, the viability or ethicality of such a system in TIO is problematic given its sensitivities. To begin with, Stack Overflow has implemented a well-crafted downvoting option, for which there is a good informational value rationale as downvoting another user’s contribution gives “you the critically important ability to distinguish between the good, the bad, and the ugly” and the ability to “tell the difference between a post that is harmless but uninteresting, and one that is actually wrong or harmful” [59]. However, TIO contributions are not pure informational entities in the way that *Stack Overflow* posts are. They are often responses inspired by lived experience and may be imbued with emotionality and personal sharing. Therefore, it is important to preclude negativity and the perceived hostility that may arise through downvoting. Furthermore, any possibility of categorizing right and wrong answers is problematic. For forums such as Stack Overflow that trade in questions requiring answers of a factual nature, this is more straightforward, though not always perfectly clear. For TIO, the nature of the problems raised, and responses given, is such that any conception of right and wrong is meaningless, highly ambiguous, or unfeasible. Although there is the possibility of implementing a voting system restricted to upvoting to promote positivity and incentivize contributions, a resulting problem could be that an implicit competitive ranking emerges.

The gamified Strengths system differs in this regard as it offers a form of individual incentivization without the issues and pressure associated with social comparison and competition. It is also not intended to be an attempt at pointsification (cases where a stock approach of gamification has been added on top of an existing system [60]) simply to generate extrinsic motivation through the collection of awards. Rather, with a dash of gamification, the Strengths points system should serve to provide users with a way to track their activity levels on the site, the skills their activity is building, and the strengths they are actualizing. Hopefully, these are goals for which the user has an intrinsic motivation to use MOST.

## Conclusions

Digital technologies, particularly computers, have been studied as persuasive technologies for at least a couple of decades now. Beyond the general aim of designing products that people want to use, there is the goal of creating interfaces and features designed to encourage certain actions or to change a person’s attitudes and behaviors. However, it has become apparent that given the commercial pressures and the race for user attention, techniques of persuasion employed by pervasive social media and networking sites are more about capitalizing our attention and generating usage that is addictive rather than usage that prioritizes well-being and is in the user’s best interest. A growing awareness of this phenomenon and the establishment of a movement to counter it have provided us with an impetus to reflect upon and analyze the MOST therapy framework and Web platform developed for online mental health interventions. We have discussed in this paper how consideration of these issues has shaped our development of the MOST system, with the intention to foster usage and engagement that is conducive rather than detrimental to mental health and well-being.

We have also had the opportunity in this paper to critically explore the notion of well-being. We contend that although hedonic or SWB is important, it is problematically limiting to confine well-being evaluations of SNSs to this type of well-being. Rather, a notion of well-being rooted in the Aristotelian conception of eudaimonia is another important dimension of well-being to consider. Although analyses of certain commercial SNSs have been restrictively conducted only in terms of SWB, given the positive psychological and therapeutic nature of the interventional MOST system, we are in a prime position to foster this sense of eudaimonic well-being.

The MOST framework is an evolving one, and as development continues on systems and features powered by tools, technologies and methodologies from artificial intelligence, ubiquitous computing, and HCI, we must remain mindful to scrutinize these developments in terms of ethical design so that user well-being remains paramount.

## Abbreviations

CHT: Center for Humane Technology
HCI: human-computer interaction
MOST: Moderated Online Social Therapy
PWB: psychological well-being
RCT: randomized controlled trial
SDT: Self-Determination Theory
SNS: social networking site
SWB: subjective well-being
TIO: Talk It Out

## References

[1] Chou HG, Edge N (2012). 'They are happier and having better lives than I am': the impact of using Facebook on perceptions of others' lives. Cyberpsychol Behav Soc Netw.

[2] Deters FG, Mehl MR (2013). Does posting Facebook status updates increase or decrease loneliness? An online social networking experiment. Soc Psychol Personal Sci.

[3] Kross E, Verduyn P, Demiralp E, Park J, Lee DS, Lin N, Shablack H, Jonides J, Ybarra O (2013). Facebook use predicts declines in subjective well-being in young adults. PLoS One.

[4] Steers MN, Wickham RE, Acitelli LK (2014). Seeing everyone else's highlight reels: how Facebook usage is linked to depressive symptoms. J Soc Clin Psychol.

[5] Verduyn P, Ybarra O, Résibois M, Jonides J, Kross E (2017). Do social network sites enhance or undermine subjective well-being? A critical review. Soc Issues Policy Rev.

[6] Frost RL, Rickwood DJ (2017). A systematic review of the mental health outcomes associated with Facebook use. Comput Human Behav.

[7] Fogg BJ (1998). Persuasive Computers: Perspectives and Research Directions. Proceedings of the SIGCHI Conference on Human Factors in Computing Systems.

[8] Erfani SS, Abedin B (2018). Impacts of the use of social network sites on users' psychological well-being: a systematic review. J Assoc Inf Sci Technol.

[9] Ryff CD, Singer BH (2008). Know thyself and become what you are: a eudaimonic approach to psychological well-being. J Happiness Stud.

[10] Huang C (2010). Internet use and psychological well-being: a meta-analysis. Cyberpsychol Behav Soc Netw.

[11] Boyd DM, Ellison NB (2007). Social network sites: definition, history, and scholarship. J Comput Mediat Commun.

[12] Fogg BJ (2003). Persuasive Technology: Using Computers To Change What We Think And Do.

[13] Fogg BJ (1999). Persuasive technologies. Commun ACM.

[14] Oinas-Kukkonen H, Harjumaa M (2009). Persuasive systems design: key issues, process model, and system features. Commun Assoc Inform Syst.

[15] (2019). Centre for Humane Technology.

[16] Centre for Humane Technology.

[17] Wayback Machine - Internet Archive.

[18] Diener E (1984). Subjective well-being. Psychological Bulletin.

[19] Diener E, Suh EM, Lucas RE, Smith HL (1999). Subjective well-being: three decades of progress. Psychol Bull.

[20] Ryan RM, Deci EL (2001). On happiness and human potentials: a review of research on hedonic and eudaimonic well-being. Annu Rev Psychol.

[21] Ryff CD, Singer BH (2006). Best news yet on the six-factor model of well-being. Soc Sci Res.

[22] Ryff CD (2014). Psychological well-being revisited: advances in the science and practice of eudaimonia. Psychother Psychosom.

[23] Peters D, Calvo RA, Ryan RM (2018). Designing for motivation, engagement and wellbeing in digital experience. Front Psychol.

[24] D'Alfonso S, Santesteban-Echarri O, Rice S, Wadley G, Lederman R, Miles C, Gleeson J, Alvarez-Jimenez M (2017). Artificial intelligence-assisted online social therapy for youth mental health. Front Psychol.

[25] Alvarez-Jimenez M, Bendall S, Lederman R, Wadley G, Chinnery G, Vargas S, Larkin M, Killackey E, McGorry P, Gleeson J (2013). On the HORYZON: moderated online social therapy for long-term recovery in first episode psychosis. Schizophr Res.

[26] Lederman R, Wadley G, Gleeson J, Bendall S, Álvarez-Jiménez M (2014). Moderated online social therapy: designing and evaluating technology for mental health. ACM Trans Comput-Hum Interact.

[27] Peterson C (2006). A Primer in Positive Psychology.

[28] Seligman ME, Csikszentmihalyi M (2000). Positive psychology: An introduction. Am Psychol.

[29] Wadley G, Lederman R, Gleeson J, Alvarez-Jimenez M (2013). Participatory Design of an Online Therapy for Youth Mental Health. Proceedings of the 25th Australian Computer-Human Interaction Conference: Augmentation, Application, Innovation, Collaboration.

[30] Gleeson J, Lederman R, Koval P, Wadley G, Bendall S, Cotton S, Herrman H, Crisp K, Alvarez-Jimenez M (2017). Moderated online social therapy: a model for reducing stress in carers of young people diagnosed with mental health disorders. Front Psychol.

[31] Calvo RA, Peters D (2014). Positive Computing: Technology for Wellbeing and Human Potential.

[32] Rice S, Gleeson J, Davey C, Hetrick S, Parker A, Lederman R, Wadley G, Murray G, Herrman H, Chambers R, Russon P, Miles C, D'Alfonso S, Thurley M, Chinnery G, Gilbertson T, Eleftheriadis D, Barlow E, Cagliarini D, Toh J, McAlpine S, Koval P, Bendall S, Jansen JE, Hamilton M, McGorry P, Alvarez-Jimenez M (2018). Moderated online social therapy for depression relapse prevention in young people: pilot study of a 'next generation' online intervention. Early Interv Psychiatry.

[33] Alvarez-Jimenez M, Gleeson J, Bendall S, Penn D, Yung A, Ryan R, Eleftheriadis D, D'Alfonso S, Rice S, Miles C, Russon P, Lederman R, Chambers R, Gonzalez-Blanch C, Lim M, Killackey E, McGorry P, Nelson B (2018). Enhancing social functioning in young people at Ultra High Risk (UHR) for psychosis: a pilot study of a novel strengths and mindfulness-based online social therapy. Schizophr Res.

[34] Gleeson J, Lederman R, Herrman H, Koval P, Eleftheriadis D, Bendall S, Cotton SM, Alvarez-Jimenez M (2017). Moderated online social therapy for carers of young people recovering from first-episode psychosis: study protocol for a randomised controlled trial. Trials.

[35] Alvarez-Jimenez M, Bendall S, Koval P, Rice S, Cagliarini D, Valentine L, D'Alfonso S, Miles C, Russon P, Penn DL, Phillips J, Lederman R, Wadley G, Killackey E, Santesteban-Echarri O, Mihalopoulos C, Herrman H, Gonzalez-Blanch C, Gilbertson T, Lal S, Chambers R, Daglas-Georgiou R, Latorre C, Cotton SM, McGorry PD, Gleeson JF (2019). HORYZONS trial: protocol for a randomised controlled trial of a moderated online social therapy to maintain treatment effects from first-episode psychosis services. BMJ Open.

[36] headspace National Youth Mental Health Foundation.

[37] Rice S, Gleeson J, Leicester S, Bendall S, D'Alfonso S, Gilbertson T, Killackey E, Parker A, Lederman R, Wadley G, Santesteban-Echarri O, Pryor I, Mawren D, Ratheesh A, Alvarez-Jimenez M (2018). Implementation of the enhanced Moderated Online Social Therapy (MOST+) model within a national youth e-Mental health service (eheadspace): protocol for a single group pilot study for help-seeking young people. JMIR Res Protoc.

[38] van Gemert-Pijnen JE, Kelders SM, Bohlmeijer ET (2014). Understanding the usage of content in a mental health intervention for depression: an analysis of log data. J Med Internet Res.

[39] Peterson C, Seligman M (2004). Character Strengths and Virtues: A Handbook and Classification.

[40] Seligman ME (2002). Authentic Happiness: Using the New Positive Psychology to Realize Your Potential for Lasting Fulfillment.

[41] Nezu AM, Nezu CM, D'Zurilla T (2013). Problem-Solving Therapy: A Treatment Manual.

[42] Kuss DJ, Griffiths MD (2011). Online social networking and addiction--a review of the psychological literature. Int J Environ Res Public Health.

[43] Kuss D, Griffiths M (2017). Social networking sites and addiction: ten lessons learned. Int J Environ Res Public Health.

[44] Wozney L, Huguet A, Bennett K, Radomski AD, Hartling L, Dyson M, McGrath PJ, Newton AS (2017). How do eHealth programs for adolescents with depression work? A realist review of persuasive system design components in internet-based psychological therapies. J Med Internet Res.

[45] Nahum-Shani I, Smith SN, Spring BJ, Collins LM, Witkiewitz K, Tewari A, Murphy SA (2018). Just-in-Time Adaptive Interventions (JITAIs) in mobile health: Key components and design principles for ongoing health behavior support. Ann Behav Med.

[46] D'Alfonso S, Carpenter N, Alvarez-Jimenez M (2018). Making the MOST Out of Smartphone Opportunities for Mental Health. Proceedings of the 30th Australian Conference on Computer-Human Interaction.

[47] Deterding S, Sicart M, Nacke L, O'Hara K, Dixon D (2011). Gamification. Using Game-design Elements in Non-gaming Contexts. Proceedings of the Extended Abstracts on Human Factors in Computing Systems.

[48] Llagostera E (2012). On Gamification and Persuasion. Proceedings of SBGames 2012.

[49] Hamari J, Koivisto J (2013). Social Motivations To Use Gamification: An Empirical Study Of Gamifying Exercise. Proceedings of the 21st European Conference on Information Systems.

[50] Selinger E, Sadowski J, Seager T, Walz SP, Deterding S (2014). Gamification and morality. The Gameful World: Approaches, Issues, Applications.

[51] Fleming TM, Bavin L, Stasiak K, Hermansson-Webb E, Merry SN, Cheek C, Lucassen M, Lau HM, Pollmuller B, Hetrick S (2016). Serious games and gamification for mental health: current status and promising directions. Front Psychiatry.

[52] Brown M, O'Neill N, van Woerden H, Eslambolchilar P, Jones M, John A (2016). Gamification and adherence to web-based mental health interventions: a systematic review. JMIR Ment Health.

[53] Lister C, West JH, Cannon B, Sax T, Brodegard D (2014). Just a fad? Gamification in health and fitness apps. JMIR Serious Games.

[54] Pereira P, Duarte E, Rebelo F, Noriega P, Marcus A (2014). A review of gamification for health-related contexts. Design, User Experience, and Usability. User Experience Design for Diverse Interaction Platforms and Environments.

[55] Kim TW, Werbach K (2016). More than just a game: ethical issues in gamification. Ethics Inf Technol.

[56] Cavusoglu H, Zhuolun L, Huang K (2015). Can Gamification Motivate Voluntary Contributions?: The Case of StackOverflow Q&A Community. Proceedings of the 18th ACM Conference Companion on Computer Supported Cooperative Work & Social Computing.

[57] Bornfeld B, Rafaeli S (2017). Gamifying with badges: a big data natural experiment on Stack Exchange. First Monday.

[58] (2017). FeverBee.

[59] Atwood J (2009). Stack Overflow.

[60] Knutas A, Lee N (2018). Gamification. Encyclopedia of Computer Graphics and Games.

